# Impact of Combined Lentectomy, Vitrectomy, and Trabeculectomy on Secondary Glaucoma due to Lens Dislocation

**DOI:** 10.1155/joph/6057819

**Published:** 2025-10-29

**Authors:** Yuxin Zhao, Zhaoxia Wang, Jing Liu, Shanshan Yue

**Affiliations:** ^1^Department of Ophthalmology, Weihai Central Hospital Affiliated to Qingdao University, Weihai, Shandong, China; ^2^Department of Pediatrics, Weihai Central Hospital Affiliated to Qingdao University, Weihai, Shandong, China

**Keywords:** lentectomy, trabeculectomy, traumatic glaucoma, vitrectomy

## Abstract

**Objective:**

This study aims to evaluate the clinical effectiveness of combining lentectomy, vitrectomy, and trabeculectomy in treating secondary glaucoma caused by lens dislocation.

**Methods:**

We retrospectively analyzed data from 20 cases (20 eyes) of traumatic lens dislocation accompanied by secondary glaucoma. The patients underwent standard 3-port pars plana vitrectomy and the upper trabeculectomy. Mitomycin C (MMC) was applied below scleral flap during surgery. We evaluated preoperative and postoperative visual acuity, intraocular pressure (IOP), and any complications.

**Results:**

Postsurgery, visual acuity improved in 18 of the 20 eyes (90%). The operation was considered successful if postsurgery, the use of topical antiglaucoma drugs was no longer necessary or reduced, and the IOP was controlled within the range of 6–21 mmHg. Of the cases studied, 18 eyes (90%) had a successful outcome, while 2 eyes (10%) were unsuccessful, with an IOP of less than 6 mmHg. All 20 eyes (100%) developed follicles postoperatively. Complications included intraocular hemorrhage in 3 eyes, low intraocular pressure in 6 eyes, choroidal detachment in 4 eyes, and hyperfiltration in 2 eyes.

**Conclusion:**

The combination of lentectomy, vitrectomy, and trabeculectomy presents a safe and effective method for reducing intraocular pressure and improving visual acuity in patients with secondary glaucoma resulting from lens dislocation.

## 1. Introduction

The secondary glaucoma caused by the traumatic lens dislocation is a significant complication of ocular trauma, and its complex pathogenesis makes treatment challenging [1, 2]. Between June 2021 and December 2022, the ophthalmology department of our hospital addressed this issue by performing a combined lentectomy, vitrectomy, and trabeculectomy on 20 patients with secondary glaucoma due to traumatic lens dislocation in 20 eyes. A 3-month postoperative follow-up indicated satisfactory outcomes. We detail these results in the following report.

## 2. Methods and Materials

Case selection criteria: all patients have a history of trauma; intraocular pressure > 30 mmHg, intraocular pressure cannot be controlled normally after 5–7 days of treatment with antiglaucoma drugs; dislocation of the lens; and nuclear hardness of lens ≤ Grade III. Exclusion criteria: lens dislocation secondary to glaucoma with no history of trauma; complete dislocation of the lens into the anterior chamber; nuclear hardness of lens > Grade III, cases of unbreakable nucleus by ultrasonic crushing; and cases combined with retinal detachment.

1. General information: there were 20 patients (20 eyes) with secondary glaucoma due to traumatic lens dislocation, including 17 males and 3 females, with an age range of 17–62 years, and an average of (46.00 ± 12.07) years. The duration of the disease ranged from 4 to 20 days. Preoperative visual acuity was determined from light perception to 0.05. Preoperative visual acuity and anterior segment conditions were as follows: 16 eyes had partial crystalline lens dislocation, 4 eyes had complete dislocation, all of which had entered the vitreous cavity, anterior chamber or vitreous hemorrhage in 7 eyes, traumatic cataract in 13 eyes, with 9 eyes having nuclear hardness below Grade III, and 4 eyes having Grade III nucleus. None of the cases had retinal detachment, and the pupil was larger than 6 mm (traumatic mydriasis). Causes of injury: 16 eyes with blunt trauma, including 8 eyes injured by stones, 3 eyes hit by punches, 3 eyes hurt by tree branches, and 2 eyes by falls or collisions. The remaining 4 eyes were injured by explosion. IOP was measured using a noncontact tonometer. IOP ranged from 32.00 to 74.00 mmHg, with an average of 54.50 ± 11.18 mmHg (1 mmHg = 0.133 kPa). All patients were treated with 20% mannitol dropping, oral acetazolamide, and 0.5% timolol dropping for 5–7 days, but the intraocular pressure could not be controlled normally. Due to the high intraocular pressure and corneal edema on admission, the anterior chamber angle could not be clearly examined. After using intraocular pressure-lowering drugs, 8 patients were able to have their anterior chamber angles examined, with 4 eyes showing angle contusions, 2 eyes with narrowed angles, and 2 eyes with angle adhesions.
2. Surgical methods: first, 23-gauge three-port pars plana vitrectomy is made through the flat part of the ciliary body. Then, intracapsular cutting (or ultrasonic grinding) of the lens and cutting of the vitreous, especially the vitreous in the anterior chamber and at the internal filtration site, are performed. For patients with lens dislocation into the vitreous cavity, the core part of the vitreous is cut first, perfluorocarbon is injected to make the lens float upwards, keeping it at a certain distance from the retina. Then, the lens is cut (or ultrasonic grinding), the intraocular perfluorocarbon is replaced, and the three-port incision is closed. Above the cornea, a 4 × 4-mm scleral flap with the corneal limbus as its base, about half the thickness of the sclera, is implanted. Underneath the flap, a cotton piece soaked in 0.2 mg/mL mitomycin C (MMC) is placed for 5 min. The cotton piece is removed, thoroughly rinsed with 100 mL of BSS solution, and then trabeculectomy and peripheral iridectomy under the scleral flap are performed. Scleral incisions were closed with 7-0 absorbable sutures; conjunctival incisions of the scleral flap and filtering bleb were closed with 10-0 nylon sutures. None of the cases had an artificial lens implanted.

## 3. Results

1. Postoperative corrected visual acuity: in 20 eyes of 20 cases, postoperative corrected visual acuity improved in 18 eyes and remained unchanged in 2 eyes (Table 1).
2. Intraocular pressure: the average intraocular pressure of 20 patients before surgery was 54.50 ± 11.18 mmHg (range from 32.00 to 74.00 mmHg). After surgery, the average intraocular pressure was 12.10 ± 6.48 mmHg (range from 5.60 to 18.50 mmHg) significantly decreased (*p* < 0.05). If, after 3 months postoperatively, no antiglaucoma medications are required, or only local antiglaucoma medications are needed, and the IOP is between 6 and 21 mmHg, the surgery is considered successful. After an average follow-up period of 3 months for the patients, 18 eyes were successful, accounting for 90%; 2 eyes were unsuccessful, with IOP in both eyes < 6 mmHg.
3. Filtration bleb: in 20 eyes, 15 eyes (75%) formed typical filtration blebs and 5 eyes (25%) formed flat filtration blebs.
4. Complications: postoperative intraocular hemorrhage occurred in 4 eyes and was absorbed within 3–7 days after surgery. Low intraocular pressure was observed in 6 eyes, including 4 eyes with choroidal detachment, and were absorbed and reattached within 7 days through using of methylprednisolone sodium succinate and 20% mannitol. Overfiltration was observed in 2 eyes, which returned to normal intraocular pressure after pressure bandaging for 3–5 days.

## 4. Discussion

### 4.1. The Pathogenesis of Secondary Glaucoma Following Traumatic Dislocation of the Crystalline Lens

The pathogenesis of secondary glaucoma following traumatic dislocation of the crystalline lens is quite complex and is associated with factors such as vitreous in the anterior chamber, vitreous pupillary block, and vitreous zonular obstruction. When the vitreous enters the anterior chamber, it directly blocks the angle of the anterior chamber, leading to an obstruction in the outflow of aqueous humor. This obstruction results in an increase in intraocular pressure. The presence of vitreous in the anterior chamber can also cause pupillary block, where aqueous humor cannot flow smoothly from the posterior chamber to the anterior chamber. This creates a pressure difference between the anterior and posterior chambers, pushing the iris forward, causing a shallow anterior chamber. This, in turn, further impairs the drainage function of the angle of the anterior chamber. When the vitreous obstructs the zonules, it can lead to aqueous humor misdirection, which can result in the development of malignant glaucoma [1, 2]. Clinically, one or several of these factors may be present simultaneously in cases of secondary glaucoma following traumatic dislocation of the crystalline lens.

### 4.2. Surgical Approaches

With the increasing complexity of glaucoma cases in clinical practice, the role of the vitreous in the pathogenesis of complex glaucoma has become increasingly important. This has led to the rapid introduction of vitrectomy surgery into the treatment of secondary glaucoma [3, 4]. To improve the success rate of surgery, some authors have combined filtration surgery with antimetabolism drugs (such as 5-FU and MMC) or filtration surgery with anterior vitrectomy [5, 6]. Weiqun Wang et al. have achieved good results by combining filtration surgery with anterior vitrectomy and antimetabolism drugs [7]. Ming Zhou et al. treated 26 eyes with traumatic dislocation of the crystalline lens and secondary glaucoma using a three-channel closed-loop crystalline lens vitrectomy combined with trabeculectomy, also achieving satisfactory results [8]. There are fewer reports on the combined removal of the crystalline lens and vitreous, along with filtration surgery and antimetabolism drugs, especially in cases where both vitreous and partial dislocation of the crystalline lens are present in the anterior chamber. The removal of the crystalline lens is beneficial for completely clearing the vitreous in the anterior chamber, preventing mechanical obstruction of the filtration angle, and the use of MMC can reduce the formation of conjunctival and scleral flap scars, both of which contribute to improving the success rate of surgery. In this group of patients, all had significant visual impairment and secondary glaucoma, with partial or complete dislocation of the crystalline lens. Drug treatment alone was not effective in controlling intraocular pressure. Therefore, lens vitrectomy combined with trabeculectomy and the use of MMC were performed in all cases, and satisfactory results were achieved.

### 4.3. Surgical Complication

Complications of crystalline lens vitrectomy combined with trabeculectomy surgery may vary in different reports. In a report by Xiao Chen et al. of 18 cases with 18 eyes treated with vitrectomy through the flat part of the ciliary body combined with filtration surgery for glaucoma without a crystalline lens or artificial lens, there were 2 cases with minimal anterior chamber hemorrhage postoperatively and 1 case with long-term hypotony [9]. Weiqun Wang et al. reported cases with 16 eyes treated with vitrectomy combined with filtration surgery and MMC for secondary glaucoma without a crystalline lens or artificial lens. Early postoperative complications included conjunctival wound leakage in 1 case, corneal epithelial staining in 2 cases, anterior chamber hemorrhage in 3 cases, uveitis reaction in 3 cases, and hypotony in 3 cases. The main long-term complication was low intraocular pressure in 1 case [7]. In the cases mentioned in this text, postoperative complications included intraocular hemorrhage in 4 cases, hypotony in 6 cases, including 4 cases with choroidal detachment, and overfiltration in 2 cases. The possible reasons for these complications could be attributed to the fact that the preoperative intraocular pressure was relatively high in this group of patients, which could not be controlled with medication. During and after surgery, there was a significant reduction in intraocular pressure, leading to increased capillary leakage from the choroid. Therefore, it can be observed that during the vitrectomy through the flat part of the ciliary body with a three-channel closed-loop approach, maintaining good intraocular pressure resulted in a significant reduction in intraoperative and postoperative complications, thus avoiding complications such as choroidal effusion. During the surgical procedure, when the crystalline lens is removed, the cloudy vitreous is also removed, which has the advantage of fewer postoperative complications and better visual recovery.

In conclusion, the pathogenesis of secondary glaucoma due to crystalline lens dislocation is complex, and the failure rate of conventional filtration surgeries is relatively high. Crystalline lens vitrectomy combined with trabeculectomy is a relatively safe and effective method for treating this type of glaucoma, especially in cases where the vitreous enters the anterior chamber, there is pupillary block due to the crystalline lens and vitreous, and there is zonular obstruction by the vitreous.

## Figures and Tables

**Table 1:** Preoperative and postoperative visual acuities (final visit).

Visual acuity	Preoperatively *N* (%)	Postoperatively *N* (%)
Light perception∼hand movements	17 (85)	2 (10)
Counting fingers∼0.05	3 (15)	1 (5)
0.06∼0.1	0	2 (10)
0.12∼0.3	0	10 (50)
0.4∼0.5	0	5 (25)
0.6∼1.0	0	0

## Data Availability

The datasets used and/or analyzed during the current study are available from the corresponding author upon reasonable request.
